# Transition behaviors of γ–β_0_/β in V-, Cr-, Mn-doped TiAl alloys

**DOI:** 10.1038/s41598-021-89273-6

**Published:** 2021-05-06

**Authors:** Lin Zhu, Hui-Chun Xue, Shu-Xin Yao, Lin Li

**Affiliations:** College of Sciences, Northeastern University, Shenyang, 110819 China

**Keywords:** Metals and alloys, Condensed-matter physics

## Abstract

The behavior of γ–β/β_0_ phase transition in TiAl alloy doped with β stabilizers (V, Cr, Mn) are studied by using the first principles method. It is found that alloying addition as well as anharmonic lattice vibration and disordered atomic occupation contributes to enhance the stability of cubic structure and accordingly introduce the disordered β phase into the high-temperature microstructure. The formation of low-temperature β_0_ phase originates from not only the stabilization of cubic structure but also the destabilization of tetragonal structure. In particular, the latter is the main reason for the premature precipitation of the hard-brittle β_0_ phase in the room-temperature microstructure at low nominal doping concentrations. We also find a special doping region in which the γ and the β phases are stable, while the β_0_ phase is unstable. The existence of this region provides an opportunity for the regulation of the contents of β and β_0_ phases.

## Introduction

Titanium aluminides are promising light-weight structural materials for high-temperature applications in aerospace industries, owing to their good oxidation resistance, specific yield strength and specific stiffness^1^. In their microstructures there mainly exists two components, a face-centered tetragonal L1_0_ structure (space group P4/mmm, γ phase) and a hexagonal D0_19_ structure (space group P6_3_/mmc, α_2_ phase)^2^. The low symmetries of these phases impose several restrictions to dislocation glide, resulting in low ductility of the alloys. When the material is deformed at a high strain rate, plastic deformability decreases greatly even at high temperatures. In such a context, the conventional thermo-mechanical process is hard to perform^3^.

The disordered cubic-structured β phase (space group ${\text{Im}} \overline{3}{\text{m}}$, A2 structure) can provide sufficient independent slip systems during deformation^4^. With the addition of the so-called β stabilizers, such as V, Cr, Mn, Nb, Mo and W, this phase can be introduced into the high-temperature microstructure, thus improving the thermoplastic formability of the γ-TiAl based alloys. Tetsui et al.^5,6^ reported that adding 5 at.% Mn to Ti–42Al can drive the microstructure into α + β region at 1573 K, which makes the alloy exhibit superior hot forging performance. Takayama et al.^7^ also observed 20 vol% β phase in the microstructure of Ti–42Al–8 V quenched at 1473 K. Nakashima et al.^8^ summarized that the critical addition of V, Cr, Nb, Mo to precipitate the β phase in the same alloy system at 1473 K were 8, 4, 9, 2 at.%, respectively. However, the addition often results in the precipitation of the ordered hard-brittle β_0_ phase (space group ${\text{Pm}}\overline{3}{\text{m}}$, B2 structure), at low temperature^9^. It was observed that about 6 vol% β_0_ phase was formed in Ti–42Al–5Mn at room temperature^10^. According to the isothermal sections at 1273 K concluded by Kanuima et al.^11^, the β_0_ phase starts to occur in Ti–42Al when the amounts of V, Cr, Mn, Nb, Mo, W reach about 5, 2, 3, 8.5, 1, 1 at.% respectively. For Ti–48Al system, the β_0_ phase was observed by Sun et al.^12^ as the addition of V, Cr, Fe, Nb arrived at 4, 2, 1, 6 at.% respectively; the β_0_ phase were found to increase significantly with the increase of addition, deteriorating the yield and ultimate tensile strength, tensile ductility, as well as creep-rupture life. The above-mentioned studies indicates that the precipitation of β and β_0_ phases in TiAl alloy is closely correlated to many factors. They may include alloying element, temperature, and Al content etc. Using density functional theory (DFT) calculations, Hu et al.^13^ predicted that the energetically favored β_0_ phase can be formed when Al was substituted by 10.50 at.% W and 11.50 at.% Mo, respectively. Ruan et al.^14^ used DFT method combined with virtual crystal approximation (VCA) to give the Cr contents of 10–15 at.% and 9–20 at.% for stabilizing the β_0_ phase in Ti_0.5_Al_0.5−x_Cr_x_ and Ti-riched Ti_0.56_Al_0.44−x_Cr_x_, respectively. It is interesting to note that the calculated critical addition of β stabilizers for stabilizing the β_0_ phase is much lower than those for precipitating that phase in the alloy. This implies that the behavior of γ–β/β_0_ phase transition is highly complicated, which issues a great challenge for the reasonable control of the β and β_0_ contents, and the modification of TiAl alloy.

In practice, heat-treatment techniques including solid solution, aging and cooling processes, used to be applied to reduce the β_0_ fraction^7,10,15,16^. Another way is to introduce the empirical equivalent method to composition design. With respect to the maximum solid solution strengthening, Sun et al.^12^ summarized an empirical equation of the Cr equivalent for Ti–48Al. Kong et al.^17^ proposed the room- and high-temperature Mo equivalents for Ti–43Al, used to control the β_0_ and β fractions, respectively. In order to achieve better modification of the alloy, a further understanding on the behavior of γ–β/β_0_ structural phase transition is still needed.

In this paper, DFT method is applied to investigate the phase transitions of V-, Cr-, and Mn-doped TiAl alloys. Formation and cohesive energies are calculated to examine the site preference of these β stabilizers. Phonon spectra and elastic constants are calculated to focus on the transition behaviors under alloying, temperature, and atomic disorder. It is expected to provide some useful information for the control of the phase compositions of β-stabilized TiAl alloys.

## Results

There still exists some controversy on the site preferences of the β stabilizers. Hao et al.^18^ experimentally reported that Zr, Nb, and Ta tended to substitute Ti, while the site preferences of V, Cr, and Mn changed considerably with Ti/Al ratio. Reviere et al.^19^, however, proposed that Cr slightly favored Al site and Mn located randomly in Al-rich lattice. For the site preference, theoretical studies based on γ- and β_0_-type structures often gave opposite results^13,20^. Even for γ phase, V, Cr, Nb, Mo, Ta, W were found to preferentially substitute for Al in Ref.^21^, while they were favored Ti sites in other reports^13,20^. To examine the site preference of X (X = V, Cr, Mn), formation energy (${E}_{form}$) and cohesive energy (${E}_{coh}$) are calculated. They are defined by the following equations:

1 $${E}_{form}=({E}_{tot}-{N}_{Ti}{E}_{solid}^{Ti}-{N}_{Al}{E}_{solid}^{Al}-{N}_{X}{E}_{solid}^{X})/({N}_{Ti}+{N}_{Al}+{N}_{X})$$

2 $${E}_{coh}=({E}_{tot}-{N}_{Ti}{E}_{atom}^{Ti}-{N}_{Al}{E}_{atom}^{Al}-{N}_{X}{E}_{atom}^{X})/({N}_{Ti}+{N}_{Al}+{N}_{X})$$

where ${E}_{tot}$ is the total energy of supercell with one atom replaced by X; ${N}_{X}$ represents the number of a specific atom; ${E}_{solid}^{X}$ denotes the energy per atom of pure constituents in the solid states; ${E}_{atom}^{X}$ refers to the energy per isolated atom.

**Figure 1:**
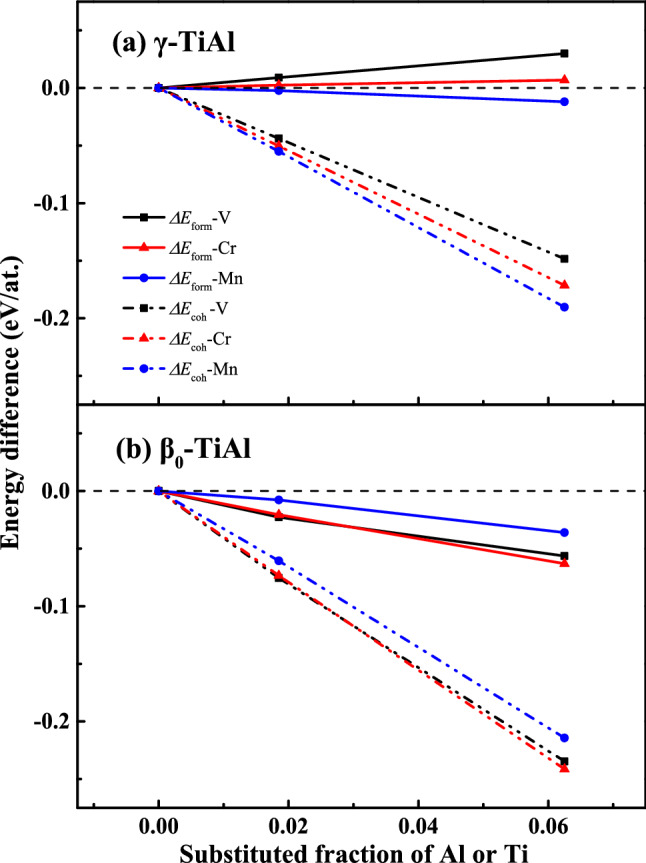
Calculated formation *E*_form_ and energies *E*_coh_ of X = V, Cr, and Mn sitting at Al and Ti sites in γ (**a**) and β_0_ (**b**) phases as a function of composition. The relative energy *ΔE* denotes the energy difference between Al-site and Ti-site, and positive (negative) value means that Ti (Al) site is energetically favored. To simulate the low level doping 3 × 3 × 3 (1.825 at.%) and 2 × 2 × 2 (6.25 at.%) supercells are constructed with the central atom replaced by X.

Figure 1 shows the calculated relative formation and cohesive energies with Ti and Al sublattices occupied by X. Formation energies suggest that V and Cr tend to locate at Ti site while Mn prefers to occupy Al site in the γ-type structure as shown in Fig. 1a. Moreover, the site preference increases with the increase of doping concentration. Cohesive energies, however, deliver a distinct result in which Al-sublattice is energetically favored for all these β stabilizers. Such a difference is not surprising, since formation energy usually reflects the alloying ability and cohesive energy describes the stability of crystal structure. It is clear that X sitting at Al site leads to higher structural stability, which agrees with those of previous calculations^21–23^. Interestingly, both energies illustrated in Fig. 1b gives the qualitatively consistent results for the β_0_-type structure.

**Figure 2:**
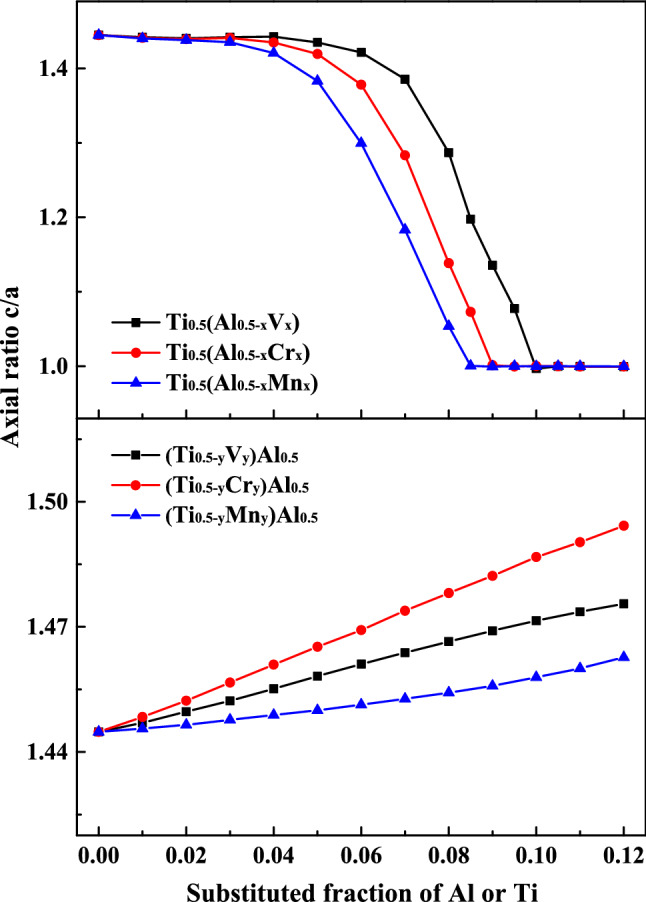
Axial ratios (c/a) as a function of composition, Ti_0.5_(Al_0.5−x_X _x_) (X = V, Cr, and Mn; x = 0–0.12) (top), (Ti_0.5-y_X _y_)Al_0.5_ (X = V, Cr, and Mn; y = 0–0.12) (bottom).

Figure 2 plots the doping dependent axial ratios (c/a) of γ-type Ti–Al–X systems. When Al is partially replaced, the ratio decreases with the increase of X, and down towards unity at the addition of 10 at.% V, 9 at.% Cr, and 8.5 at.% Mn, respectively. By contrast, those ratios increase, and the structures become slightly more anisotropic when Ti is partially substituted. These results also indicate to some extent the importance of Al-site doping for the transition from tetragonal to cubic structure.

**Figure 3:**
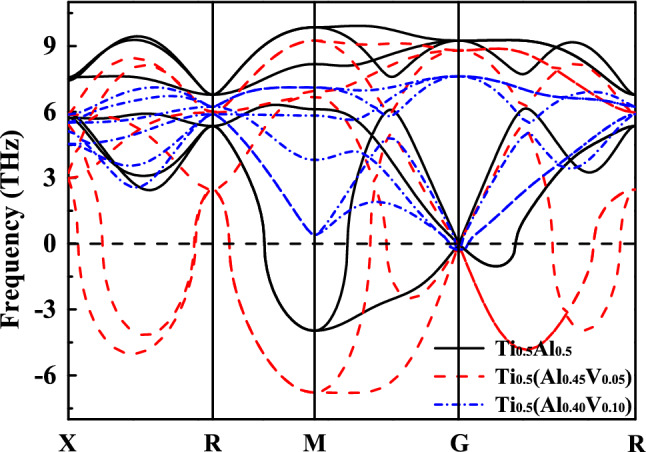
Composition dependent phonon dispersion curves of β_0_-type Ti–Al–V at zero temperature.

Figure 3 depicts the phonon dispersion curves of the β_0_-type Ti–Al–V system. From the viewpoint of lattice dynamics, the phonon frequencies of a stable structure should be positive throughout the entire Brillion zone. However, the acoustic phonons of β_0_-Ti_0.5_Al_0.5_ show obvious imaginary frequencies along the paths of R–M, G–M, and G–R. It suggests that the restoring forces are insufficient to stabilize the atoms at their equilibrium positions. Therefore, the cubic structure is in an unstable equilibrium state. The maximum imaginary frequencies occur at the high symmetric M (0.5 0.5 0.0) point, thus the phonon modes at this point are the most significant factor leading to the structural instability. Polarization vectors show that the soft phonon mode associates to the instabilities of Ti and Al vibrations in the XY ([100]–[010]) plane. Driven by the soft mode, the structure will transform into low-symmetric ones.

With the increase of V, the imaginary frequencies at M point and on G-R path are observed to increase firstly and then decrease. When the doping level is up to as high as 10 at.%, the imaginary frequencies disappear completely, and the structure achieves a dynamically stable state. The phonon spectra of Ti–Al–Cr and Ti–Al–Mn are provided in Fig. S1 in the electronic supplementary material. The calculated critical concentrations for Cr and Mn are 9 and 8.5 at.% respectively, lower than that of V. As alloying additions, Cr and Mn show strong ability with respect to the stabilization of the cubic structure.

**Figure 4:**
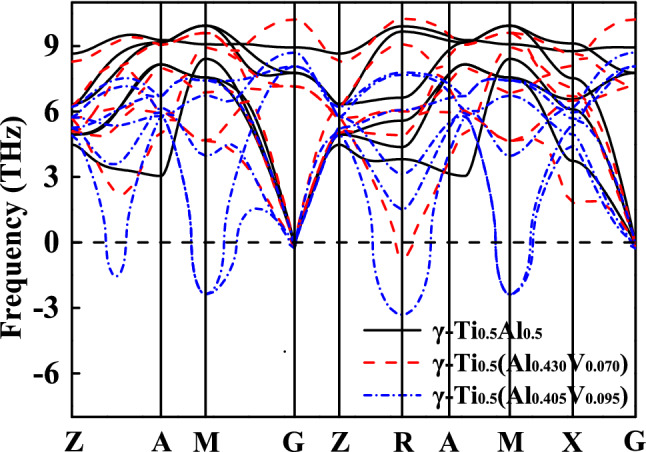
Phonon dispersion curves of γ-type TiAl and unstable Ti–Al–V at zero temperature.

The addition of β stabilizers promotes the stabilization of cubic structure, but also reduces the stability of tetragonal phase (see Figs. 4 and Fig. S2). When the concentrations of V, Cr and Mn are greater than or equal to 7.0 at.%, 6.0 at.%, 5.5 at.% respectively, imaginary frequencies are observed to appear successively at R point, M point and Z–A path of Brillouin zone. However, it is worth noting that the doping threshold for the destabilization of tetragonal phase is lower than that of the stabilization of cubic one. Therefore, the tetragonal phase with nominal composition between the two thresholds probably separate into stable tetragonal phase with poor β stabilizers and cubic phase with rich β stabilizers. This is also one of the reasons for the precipitation of the β_0_ phase at low doping concentration.

**Figure 5:**
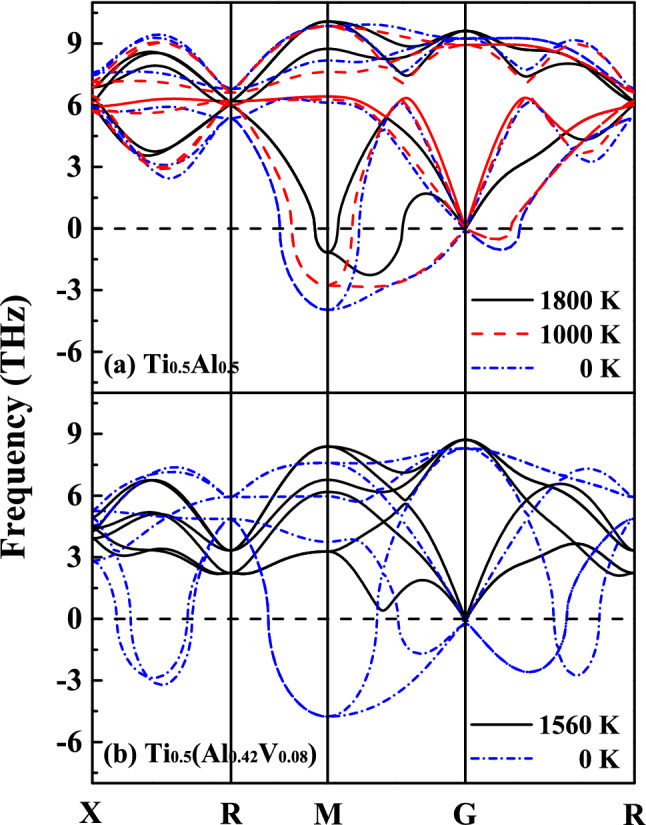
Temperature dependent phonon dispersion curves of β_0_-type Ti_0.5_Al_0.5_ (**a**) and Ti_0.5_(Al_0.42_V_0.08_) (**b**).

At low temperatures, the atomic vibrations correspond to simple harmonic motion about the positions of minimum energy. As the temperature increases, they become increasingly anharmonic, which probably changes the phonon modes and accordingly the structural stability. Finite-temperature phonon calculations are therefore performed to explore the effects of temperature on the structural stabilities of the β_0_-type Ti–Al–X. The upper temperature limit adopted in our calculations is set at 1800 K, which approaches the melting point of TiAl^24^. Generally, temperature exerts a great influence on the acoustic phonons. As shown in Fig. 5a, the imaginary frequencies of β_0_-Ti_0.5_Al_0.5_ decrease from 3.96 to 1.17 THz at M point and they disappear on G-R path at 1800 K. The stability is significantly enhanced by the temperature and the anharmonic effect from the viewpoint of lattice dynamics. Moreover, the maximum imaginary frequency deviates from the high symmetric point along the G-R path, indicating that the symmetry of the corresponding stable structure is further reduced.

Although the phonon–phonon interaction cannot fully stabilize the cubic structured Ti_0.5_Al_0.5_ at elevated temperatures, it may still play an active role in the γ–β/β_0_ phase transition. Figure 5b shows the phonon spectra of Ti_0.5_(Al_0.42_V_0.08_) at zero and elevated temperatures. The imaginary frequencies of the V-doped system almost distribute in the whole Brillouin zone at zero temperature. The structural stability is presented to be slightly higher than that of Ti_0.5_(Al_0.45_V_0.05_) (see Fig. 3) but lower than that of Ti_0.5_Al_0.5_. Generally, the β-stabilizers are heavier than both Ti and Al atoms. They should vibrate mainly in the low-frequency range, which also provides an opportunity for the hardening of acoustic phonons and structural phase transitions induced by temperature. When the temperature is increased up to 1560 K, the system is found to achieve a dynamically stable state. It is noteworthy that the minimum addition of V decreases by 2 at.% compared with that of zero temperature. The calculated minimum additions of Cr and Mn for the stabilization of cubic phase under elevated temperatures are 7.5 and 6.5 at.%, respectively (see Fig. S3).

There exists a convergence problem of phonon calculations for the fully disordered phase in VCA calculation. Elastic constants are therefore calculated to evaluate the effect of atomic disorder on structural stability. Born and Huang^25^ proposed the necessary criteria with which a mechanically stable structure should comply. For cubic structure, it takes from

$${C}_{11}>0, {C}_{44}>0, {C}_{11}-\left|{C}_{12}\right|>0, {C}_{11}+2{C}_{12}>0$$

**Figure 6:**
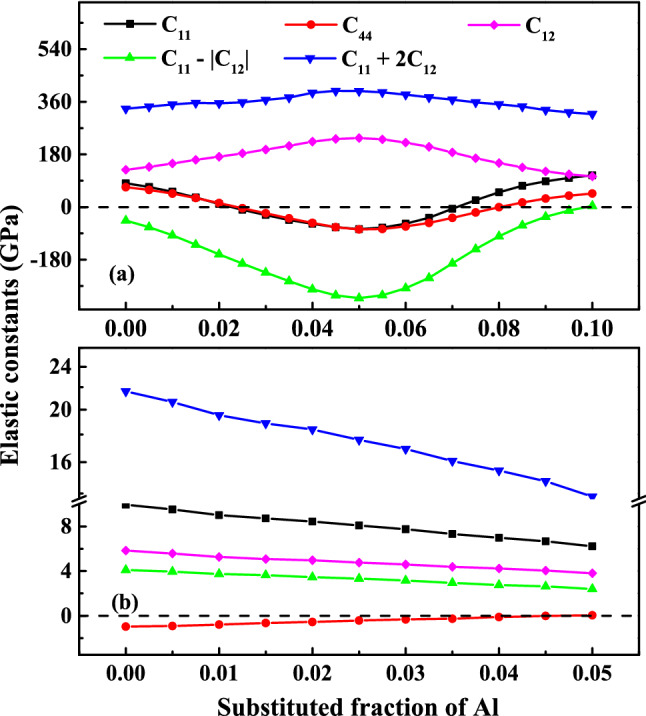
Mechanical stability criteria as a function of composition: (**a**) ordered β_0_-type Ti_0.5_(Al_0.5−x_V_x_) (x = 0–0.1), (**b**) disordered β-type Ti_0.5_(Al_0.5−y_V_y_) (y = 0–0.05).

The calculated results for β_0_- and β-Ti_0.5_(Al_0.5–x_V_x_) are diagrammatized in Fig. 6. According to the criteria, the ordered β_0_-Ti_0.5_Al_0.5_ is predicted to be mechanically unstable, as shown in Fig. 6a. The instability mainly results from ${C}_{11}-\left|{C}_{12}\right|<0$, which suggests that the shear-strain resistance is stronger than that of vertical strain in the system. With the addition of V, ${C}_{11}$ decreases firstly and then increase while ${C}_{12}$ presents an opposite trend. As a result, the criterion ${C}_{11}-\left|{C}_{12}\right|$ reaches a minimum value at 5 at.% and is greater than zero above 10 at.%. This is consistent with the variation of the structural stability identified from phonon spectra. The results of the disordered β phase are drawn in Fig. 6b. In this case, the elastic constants show significant softening. The stability of β phase only depends on the sign of ${C}_{44}$, in a sharp contrast to that of β_0_ phase. However, β-Ti_0.5_Al_0.5_ is also categorized as unstable, contrary to the simulation results based on the supercell method^26^. ${C}_{44}$ increases monotonically with the increase of V, which stabilizes the cubic structure at 5 at.%. Atomic disorder also reduces the critical concentrations of Cr and Mn to 5 and 4 at.%, respectively (see Fig. S4).

Holec et al.^20^ proposed that there may exist a special manner of combining the substitution of Al with X and the antisite of X and Ti for the doping of TiAl alloy. In their calculations, most of the considered β stabilizers prefer either the double defect (Cr, Zr, Hf) or the simple substitutional defect (V, Nb, Mo, Ta) but with insignificant energy advantage. Thus, the antisite defect is considered to increase with the increase of temperature and become one of the sources of atomic disorder besides the atomic diffusion driven by thermal fluctuation.

## Discussion

**Figure 7:**
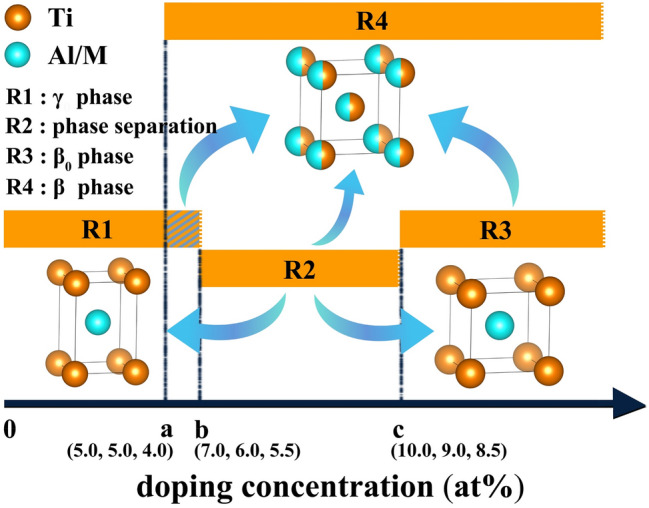
Schematic diagram of γ–β/β_0_ structural phase transition. The thresholds of R4, R2, R3 are labeled as (**a**, **b**, **c**), respectively. The ternary coordinates of these points correspond to the atomic percentage of V, Cr, Mn, respectively.

The formation of β phase depends on the stabilization of cubic structure firstly, which in turn comes from the contribution of β stabilizers and anharmonic lattice vibration at high temperature; secondly, it depends on the disorder of atomic occupation, which is also found to further enhance the stability of cubic structure. Therefore, it can be concluded that the addition of β stabilizers, the anharmonic lattice vibration and the atomic disorder create favorable conditions for the formation of β phase at high temperature. According to our calculated results, the concentrations of β stabilizers can be divided into four regions, as shown in Fig. 7. In region R1 (V < 7.0 at.%; Cr < 6.0 at.%; Mn < 5.5 at.%), the γ phase is in a stable state and β stabilizers are solubilized in it; when the amount reaches R3 (V ≥ 10.0 at.%; Cr ≥ 9.0 at.%; Mn ≥ 8.5 at.%), the cubic structured β_0_ phase enters a stable state; in R2 (7.0 at% ≤ V < 10.0 at.%; 6.0 at.% ≤ Cr < 9.0 at.%; 5.5 at.% ≤ Mn < 8.5 at.%), the γ phase is destabilized and partly transform into β_0_ phase; when the atomic occupation is fully disordered, the β phase is stable in R4 (V ≥ 5.0 at.%; Cr ≥ 5.0 at.%; Mn ≥ 4.0 at.%).

The occurrence of region R4 is important for introducing the disordered cubic β phase into the high-temperature microstructure. The stability of β phase increases with the increase of the addition and the stability of additive for cubic structure. It should be noted that the calculations on β phase were performed on the premise that the atomic occupation is in a completely disordered state. Using the elastic constants, we calculated the melting points of the alloys with the addition of 5 at.% V, Cr and Mn, and the corresponding values are 2107 K, 1951 K and 1568 K, respectively. This indicates that the disorder degree in different systems may be different at the same hot deformation temperature, e.g., 1473 K. The V- and Cr-doped system, especially the former, may require higher temperature or more additive to stabilize the β phase. In addition, the contribution from anharmonic effect is also not taken into account in the calculations of β phase. It will expand R4 slightly towards the lower doping concentration. The addition of β stabilizers not only improves the stability of β phase, but also induces the destabilization of γ phase in R2, leading to the premature precipitation of β_0_ phase at a low doping level.

Takeyama et al.^7^ reported that the microstructure of Ti-42Al-8 V contains 20 vol% β at 1473 K and a low amount of β_0_ phase at the colony boundaries at room temperature. Kainuma et al.^11^ summarized the phase equilibrium of Ti–Al-X ternary system. The phase diagrams showed that the addition of 5 at.% Cr or Mn can transform the microstructure from α phase to α + β two-phase at 1573 K, when the Ti content is fixed at 50 at%. These results are consistent with the calculated thresholds of R2 and R4 in Fig. 7. In composition design, Al content is usually reduced to promote β solidification, such as Ti–42Al^27,28^, Ti–43Al^15^, Ti–(40–45)Al^29^, Ti–(43–48)Al^30^, and Ti–48Al^31,32^. As a result, the Ti contents in these typical alloys tend to exceed 50 at.% in the microstructures. For the Ti–rich composition, a small amount of Ti occupy Al sublattice. Taking Ti–48Al as an example, we calculated the phonon curves of γ and β_0_ phases for the Cr-doped system. It can be seen from Fig. S5 that this composition further enhances the stability of cubic structure and reduces the thresholds of R2 and R3 from 6.0 at.% and 9.0 at.% to 3.0 at.% and 7.5 at.%, respectively. Hamzah et al.^32^ added 4 at.% Cr to Ti-48Al, and observed the precipitation of 2–5 vol% β_0_ phase. Their elemental analysis showed that the Ti contents in γ and β_0_ phase were about 52 at.%, and the contents of Cr were 3.79 at.% and 11.15 at.%, respectively. Sun et al.^12^ observed 9.2 vol% β_0_ phase in the same alloy system with 6 at.% V. The element contents of V in γ and β_0_ phase were determined to be 4.77 at.% and 10.0 at.%. These experimental data also basically agree with our calculated results.

Taking the thresholds of R4 and R2 as boundaries, we can determine a special region, which corresponds to the contents of V, Cr and Mn in the range of [5.0 at.%, 7.0 at.%), [5.0 at.%, 6.0 at.%), [4.0 at.%, 5.5 at.%), respectively. In this window, both the β and the γ phases are in stable states, but the β_0_ phase is unstable. It implies that controlling the amount of β stabilizers within this window can introduce a certain amount of β phase and reduce or even avoid the precipitation of β_0_ phase caused by the destabilization of γ phase and the stabilization of β_0_ phase. Kong et al.^17^ reported that the critical additions of Cr to precipitate β and β_0_ phase in Ti-43Al are 2 at.% (1473 K) and 3 at.% respectively, which actually confirmed the existence of such a region. They further found that the content of β phase increased from 3.3 to 5.0–18.8 vol% when the addition of Cr increased from 2 to 3–4 at.%; as the addition reached 3–4 at.%, no crack could be found on the surface of hot-compressed samples, and the hot deformability was improved. However, the SEM images clearly showed a large amount of β_0_ phase in the alloy with 4 at.% Cr. Although the quantitative relationship between β_0_ content and mechanical properties cannot be established presently, the excessive β_0_ phase is usually detrimental to the room-temperature plasticity and the creep resistance. For strong β stabilizers, such as Cr, the window region is too narrow (about only 1 at.%), which makes it difficult to reasonably regulate the contents of β and β_0_ phase. On the other hand, the wide window corresponds to weak β stabilizers. To obtain the stable β phase at elevated temperature, more Al atoms need to be substituted. However, the low content of Al usually increases the fraction of α_2_ phase^24,33–35^, which deteriorates the room-temperature plasticity and the high-temperature oxidation resistance. We find that the strong β stabilizers can reduce the threshold of R4, while the weak ones can increase the threshold of R2. Then the width of the window can be expanded to some extent by an appropriate combination of the two under specific Al content. In this way, the contents of β and β_0_ phase can be controlled more reasonably by adjusting composition, to seek a balance between thermoplastic and mechanical properties.

## Conclusion

The γ–β/β_0_ structural phase transitions of V-, Cr-, Mn-doped TiAl alloys are studied by first principles calculations. Al-site doping is found to have lower cohesive energy and significantly enhance the stability of the cubic structure. The effects of temperature are mainly reflected in the anharmonic lattice vibration and the disordered atomic occupation, which further promote the formation of β phase at elevated temperatures. The doping of β stabilizers not only enhances the stability of cubic structure, but also causes the destabilization of γ phase, which plays an important role in the premature precipitation of β_0_ phase from the room-temperature microstructure. In the future composition design of beta-gamma alloys, more attentions should be paid to the structural stability of β and γ phases.

## Computational details

DFT calculations are performed by using the Vienna Ab initio Simulation Package (VASP)^36,37^. Exchange–correlation functionals are treated with the generalized gradient approximation (GGA) refined by Perdew, Burke and Ernzerhof (PBE)^38^. An energy cut-off of 500 eV is used for the plane wave expansion. Fully automatic scheme is activated to generate equally spaced Γ-centered Monkhorst–Pack grids for different structures. The numbers of subdivisions along each reciprocal lattice vector are given by ${N}_{i}=\mathrm{max}\left(1, l*\left|{\overrightarrow{b}}_{i}\right|+0.5\right), i=1, 2, 3$, where ${\overrightarrow{b}}_{i}$ are the reciprocal lattice vectors and $\left|{\overrightarrow{b}}_{i}\right|$ are their norms. The parameter *l* is chosen as 40 Å.

The structural stability of the order β_0_ phase is evaluated by phonon spectra calculated with the self-consistent ab initio lattice dynamics (SCAILD) approach^39^. In this method, finite displacement technique is used to approximate the harmonic lattice vibrations at zero temperature. To simulate the thermal vibrations and their interactions, the lattice displacements are assumed to be subject to the Boltzmann statistic distribution. Both positive and negative displacements are included in the calculation procedure for improving the accuracy. Elastic constants offer qualitive assessment of the mechanical stability for the disordered structure. They are derived from the strain–stress relationship. For the phonon and elastic calculations, structural models are constructed in VCA. The potential of the virtual atom is generated by weighted averaging of the compositional elements at the doping site. The Hellmann–Feynman forces are obtained by force superposition. Recently the SCALID method combined with VCA has been successfully applied to study the temperature-induced stabilization of Ti–V and V–Cr alloy systems by Söderlind et al.^40,41^.

## Supplementary Information

Supplementary Figures.
