# Supplementary material for: Transition behaviors of γ–β0/β in V-, Cr-, Mn-doped TiAl alloys
Source: Sci Rep. 2021 May 6;11:9689. doi: 10.1038/s41598-021-89273-6 (PMC8102528; doi:10.1038/s41598-021-89273-6)
Supplement: Supplementary file 1 — Supplementary Figures. [file 41598_2021_89273_MOESM1_ESM.docx]

# Transition behaviors of γ–β_0_/β in V-, Cr-, Mn-doped TiAl alloys

Lin Zhu, Hui-Chun Xue, Shu-Xin Yao, Lin Li^*^

College of Sciences, Northeastern University, Shenyang 110819, China

Corresponding author: Lin Li

E-mail address: lilin@mail.neu.edu.cn

**Fig. S1.** Composition dependent phonon dispersion curves of β_0_-type Ti-Al-Cr and Ti-Al-Mn at zero temperature.

**Fig. S2.** Unstable phonon dispersion curves of γ-type Ti-Al-Cr and Ti-Al-Mn at zero temperature.

**Fig. S3.** Temperature dependent phonon dispersion curves of β_0_-type Ti-Al-Cr (a) and Ti-Al-Mn (b).

**Fig. S4.** Mechanical stability criteria as a function of composition, (a) ordered β_0_-type Ti_0.5_(Al_0.5-x_Cr_x_) (x = 0 – 0.1), (b) disordered β-type Ti_0.5_(Al_0.5-x_Cr _x_) (x = 0 – 0.05), (c) ordered β_0_-type Ti_0.5_(Al_0.5-x_Mn _x_) (x = 0 – 0.1), (d) disordered β-type Ti_0.5_(Al_0.5-x_Mn _x_) (x = 0 - 0.05).

**Fig. S5.** Phonon dispersion curves of γ-Ti_0.52_(Al_0.450_Cr_0.030_) (a) and β_0_- Ti_0.52_(Al_0.405_Cr_0.075_) (b).
